# The Toxic Effects and Mechanism of Acetamiprid Exposure on Testicular Tissue: ALKBH5 Regulates m^6^A Modification to Affect PI3K-AKT-Mediated Apoptosis

**DOI:** 10.3390/toxics14100903

**Published:** 2026-10-12

**Authors:** Xiaoyan Hua, Yubin Li, Lixiang Pang, Huiyuan Zhang, Yuwen Wang, Bing Wu, Haoxuan Gao, Yanan Song, Yucheng Yan, Huifang Yang, Yikai Qiu, Ji Zhao

**Affiliations:** 1 Key Laboratory of Fertility Preservation and Maintenance of Ministry of Education, Ningxia Medical University, Yinchuan 750004, China; 2 Key Laboratory of Environmental Factors and Chronic Disease Control, College of Public Health, Ningxia Medical University, Yinchuan 750004, China; 3 Department of Toxicology, Shaanxi Provincial Key Lab of Free Radical Biology and Medicine, School of Public Health, Fourth Military Medical University, Xi’an 710032, China

**Keywords:** acetamiprid, m^6^A modification, ALKBH5, PI3K-AKT, testicular injury

## Abstract

(1) Background: Acetamiprid, a widely used neonicotinoid insecticide, has increasingly drawn people’s attention to its potential reproductive toxicity risks. Although existing studies have shown that exposure to acetamiprid can cause testicular damage, its underlying molecular mechanism has not been fully elucidated. (2) Methods: This study established a mouse model of acetamiprid exposure via free drinking water and combined it with in vitro culture of TM3 cells, aiming to explore the toxic effects of acetamiprid on testicular tissue and its potential mechanism. (3) Results: Acetamiprid exposure induced significant pathological damage to the testicular tissue of mice and significantly reduced sperm quality. Further mechanism studies have shown that acetamiprid down-regulates the expression of RNA demethylase ALKBH5, leading to an overall increase in m^6^A modification levels, thereby inhibiting the activity of the PI3K/AKT signaling pathway and ultimately inducing apoptosis of TM3 cells. (4) Conclusions: In conclusion, this study, by integrating both internal and external models, has revealed a molecular mechanism by which acetamiprid is involved in testicular injury through epigenetic pathways, providing scientific basis and mechanism clues for assessing reproductive hazards caused by exposure to environmental toxins.

## 1. Introduction

Infertility is a major public health issue affecting global reproductive health. The World Health Organization reported in 2023 that approximately 15–17.5% of adults experience infertility during their lifetime—equivalent to about one in every six individuals [1]. In cases of infertility, male factors account for approximately half of the cases [2], among which oligozoospermia and asthenozoospermia are the most common. Over recent decades, sperm concentration and motility have shown a continuous declining trend in multiple countries [3], and the rate of change cannot be fully explained by genetic factors, suggesting that environmental exposure plays an important role in the decline of male reproductive function.

Environmental endocrine-disrupting chemicals are widely distributed in water bodies, soil, atmosphere, and the food chain. Even at low concentrations, their high toxicity and bioaccumulation potential pose a threat to reproductive health [4]. Pesticides represent the most commonly encountered category of these chemical substances in human exposure. Acetamiprid (ACE), a neonicotinoid insecticide, has become one of the most widely used insecticides globally due to its high selectivity for insect nicotinic acetylcholine receptors and relatively low mammalian toxicity. Its high detection rates in agricultural products, drinking water, and human urine indicate that the general population is under sustained internal exposure [5,6]. Studies have shown that ACE exposure can impair sperm fertilization capacity and embryonic developmental potential, accompanied by multiple effects including abnormal sperm function, testicular histological damage, Leydig cell toxicity, and reproductive endocrine disruption [7]. However, existing studies are largely confined to phenotypic descriptions and recordings of terminal events such as oxidative stress and apoptosis, leaving the upstream regulatory molecular mechanisms of testicular injury and sperm toxicity incompletely understood; thus, the causal chain of ACE reproductive toxicity remains to be fully elucidated.

N^6^-methyladenosine (m^6^A) is the most abundant internal modification in eukaryotic mRNA. It is catalytically deposited by the METTL3–METTL14–WTAP methyltransferase complex and dynamically removed by demethylases such as FTO and ALKBH5, regulating gene expression through effects on RNA stability, translation efficiency, and alternative splicing [8,9,10]. Spermatogenesis is highly dependent on m^6^A homeostasis: Alkbh5-deficient mice exhibit spermatogenic defects and male infertility due to aberrant alternative splicing and 3′UTR processing defects in meiotic genes [11]; ALKBH5 also maintains blood–testis barrier integrity by regulating Cdh2 mRNA translation, thereby affecting mouse fertility [12]. At the clinical level, FTO functional variants are associated with male infertility, and global m^6^A levels in spermatozoa from asthenozoospermic patients are significantly higher than those in normozoospermic fertile men, with m^6^A modification negatively correlated with sperm motility [13], suggesting that insufficient m^6^A demethylation capacity is a common molecular feature of decreased sperm quality. Notably, various environmental toxicants have been confirmed to interfere with the expression of m^6^A modification genes [14]; however, the specific mechanisms by which m^6^A demethylases participate in pesticide-induced testicular injury remain to be further elucidated.

The PI3K–AKT signaling pathway is critical for germ cell survival. Activated AKT phosphorylates the pro-apoptotic protein BAD, causing it to be sequestered by 14-3-3 proteins and preventing it from binding to the BCL-2/BCL-xL complex, thereby restoring the anti-apoptotic function of the latter and increasing the effective BCL-2/BAX ratio. Concurrently, activated AKT also inhibits Caspase-3 activity, further blocking the apoptotic process [15,16]. Previous studies have demonstrated that m^6^A modification participates in germ cell proliferation, differentiation, and apoptosis by regulating the fate of transcripts encoding PI3K–AKT pathway components or their upstream regulators [17]. This convergence suggests that the PI3K–AKT signaling pathway mediated by m^6^A demethylases may represent a critical juncture through which exogenous chemicals drive germ cell apoptosis; however, this hypothesis has not yet been validated under ACE exposure conditions.

In summary, the present study proposes the following hypothesis: ACE exposure downregulates ALKBH5, leading to elevated testicular m^6^A modification levels, which in turn disrupts the PI3K–AKT signaling pathway and apoptotic processes, ultimately resulting in testicular structural and functional damage. To test this hypothesis, we employed a mouse chronic exposure model combined with a TM3 cell model, integrated transcriptomic and m^6^A-seq analyses, and performed functional rescue experiments via ALKBH5 overexpression to systematically define the role of the ALKBH5–m^6^A–PI3K/AKT axis in ACE-induced testicular toxicity. This study aims to reveal the upstream regulatory mechanisms of neonicotinoid pesticide reproductive toxicity from the perspective of RNA epitranscriptomics and to provide molecular mechanisms and intervention targets for reproductive health risk assessment of environmental pollutants.

## 2. Materials and Methods

### 2.1. Network Toxicological Analysis

#### 2.1.1. Collection and Screening of Acetamiprid-Related Targets

Obtain the SMILE structure of acetamiprid from the PubChem database https://pubchem.ncbi.nlm.nih.gov/, accessed on 10 May 2023), import it into the SwissTarget Prediction platform (https://swisstargetprediction.ch/, species is set to “Homo sapiens”, accessed on 10 May 2023), and select “All targets.” Download and save the predicted targets with confidence score > 0.1. Search for “Acetamiprid” in the SuperPred database (https://prediction.charite.de/, accessed on 10 May 2023), and screen for targets with a confidence score > 70%. Consolidate the predicted targets from both platforms, remove duplicates, and construct a comprehensive acetamiprid target library.

#### 2.1.2. Collection and Screening of Reproductive Injury Targets

Based on the GeneCards (https://www.genecards.org/, accessed on 10 May 2023) and OMIM database (http://omim.org/, accessed on 10 May 2023), genes associated with “reproductive damage” were retrieved. Gene entries with scores above the median in both databases were merged, and duplicate genes were removed. The JVenne online tool (https://jvenn.toulouse.inrae.fr/app/index.html (accessed on 10 May 2023)) was applied to calculate the intersection of acetamiprid targets and reproductive injury-related targets, and subsequent regulatory network analyses were performed based on overlapping targets.

#### 2.1.3. Functional Enrichment Analysis

Gene Ontology (GO) and Kyoto Encyclopedia of Genes and Genomes (KEGG) pathway enrichment analyses were performed using the DAVID database (https://davidbioinformatics.nih.gov/, accessed on 11 May 2023). The bioinformatics online platform (https://www.bioinformatics.com.cn/, accessed on 11 May 2023) was utilized for visualization of enrichment outputs to interpret biological functions and signaling pathways modulated by target genes.

### 2.2. Experimental Animals and Poisoning Models

Forty-eight SPF-grade male 2-week-old C57BL/6J mice were used (animal ethics approval number: No.2022-N039, approved on 2 September 2022) were acclimatized for one week, then randomly assigned into four groups (*n* = 12 per group): control group (0 mg/kg), low-dose group (4.55 mg/kg), medium-dose group (9.10 mg/kg), and high-dose group (18.2 mg/kg). Continuous toxic exposure was administered via free drinking water for 6 months, and the exposure dosages were converted based on the acute oral LD50 value (182 mg/kg) in rats. All animal experiments were conducted at the Experimental Animal Center of Ningxia Medical University, and all procedures strictly complied with the Guidelines for Ethical Review of Laboratory Animal Welfare [18].

### 2.3. Cell Culture

TM3 cells (National Collection of Authenticated Cell Cultures, Chinese Academy of Sciences, Shanghai, China, catalog No. SCSP-5035) were cultured using a specialized culture medium in an incubator at 37 °C with 5% CO_2_. Cells in the logarithmic growth phase were treated with acetamiprid at concentrations of 0, 2, 4, and 6 mmol/L (purity ≥ 98%, Aladdin™, Shanghai, China) for 24 h. The 0 mmol/L group was the vehicle control (medium without acetamiprid). Cells were harvested after 24 h of treatment. These in vitro molar concentrations cannot be directly converted to testicular tissue concentrations in our mouse model; in vitro doses were selected based on cellular IC50 to generate graded responses for mechanistic investigation.

### 2.4. Detection of CCK-8 Cell Activity

Cells were seeded into 96-well plates at a density of 1 × 10^3^ cells per well and cultured routinely for 24 h prior to 24 h of acetamiprid exposure. A volume of 10 μL CCK-8 reagent (Nanjing Kaiji Biotechnology, Nanjing, China) was added to each well, followed by 30 min light-protected incubation at 37 °C. The absorbance at 450 nm was measured using a BioTek (Instruments, Winooski, VT, USA) microplate reader. Cell viability was calculated as a percentage of the control group. The IC_50_ was estimated as the concentration at which cell viability reached 50%, which fell between 6 and 8 mmol/L (6.17 mmol/L).

### 2.5. Cell Migration Assay

Cells were seeded into 6-well plates and cultured for 12 h before 24 h acetamiprid treatment. Three parallel scratches were made vertically in each well using sterile toothpicks. Floating detached cells were washed off with PBS buffer, and the culture medium was replaced with serum-free medium. Fixed-point imaging was performed under an inverted microscope (Nikon, Tokyo, Japan), and the migration distance of cells in each group was quantitatively analyzed.

### 2.6. Sperm Morphology Analysis

The cauda epididymidis was excised and placed in pre-warmed PBS (37 °C). The tissue was minced, incubated at 37 °C for 10–15 min, and filtered through a 40-μm nylon mesh. All analyses were completed within 30 min of collection. Sperm concentration and motility were measured using a fully automatic sperm quality analyzer (SAS-II, Saissi, Beijing, China). A 10-μL aliquot was examined at 200× magnification, and at least 200 spermatozoa per sample were classified as motile or immotile. For morphology, a 10-μL aliquot was smeared, air-dried, and stained with Diff-Quik solutions I and II (15 s each, Leagene Biotechnology, Beijing, China). At least 200 spermatozoa per animal were examined at 400× magnification. Abnormalities were classified as head, neck, or tail defects, and the abnormality rate was calculated as the percentage of abnormal spermatozoa. For each group (*n* = 12), samples from at least 6 randomly selected mice were used. Technical replicates (duplicate smears) were averaged, and biological replicates were individual mice. All analyses were performed by two blinded observers.

### 2.7. Hematoxylin and Eosin Staining (HE Staining)

Fresh mouse testicular tissues were fixed in 4% polyformaldehyde for 24 h; dehydrated with a gradient ethanol solution until transparent; embedded in paraffin and sectioned (4 μm thick); stained with hematoxylin and eosin, and then observed under a microscope (20×) for examination of testicular tissue structure.

### 2.8. Transmission Electron Microscopy Experiment

Fresh mouse testicular tissues were immersed in pre-cooled electron microscopy fixative at 4 °C for 20 min, trimmed into small tissue blocks (3 mm × 1 mm × 1 mm), and further fixed in the same fixative at 4 °C for 24 h. Gradient ethanol dehydration was performed, followed by secondary dehydration with 100% acetone. After embedding and ultra-thin section preparation, the ultrastructure of testicular tissues was visualized under a HITACHI transmission electron microscope (HITACHI, Tokyo, Japan).

### 2.9. Western Blot

Total protein was extracted from mouse testicular tissues and TM3 cells using RIPA lysis buffer (Nanjing Kaiji Biotechnology, Nanjing, China), and protein concentrations were quantified with the BCA protein quantification kit (Biyuntian Biotechnology, Shanghai, China). After protein denaturation, samples were loaded for SDS-PAGE electrophoresis to separate proteins, followed by transfer onto PVDF membranes. Membranes were blocked with rapid blocking buffer at room temperature for 1 h, then washed three times with TBST buffer (10 min per wash). Primary antibodies (GAPDH, 1:1000; ALKBH5, 1:1000; METTL3, 1:1000; METTL14, 1:1000; FTO, 1:1000; CASPASE3, 1:1000; P-PI3K, 1:1000; P-AKT, 1:1000; BAX, 1:1000; BCL-2, 1:1000, all purchased from Abcam, Cambridge, MA, USA) were incubated with membranes overnight at 4 °C. After three TBST washes, HRP-conjugated secondary antibodies (dilution 1:1000) were incubated with membranes for 1 h at room temperature. Following thorough TBST washing, chemiluminescence exposure and imaging were conducted, and the gray value of protein bands was quantitatively analyzed and plotted using ImageJ (v1.54) software.

### 2.10. Quantitative Detection of m^6^A RNA Methylation

Total RNA was extracted from mouse testicular tissues and TM3 cells, and RNA purity was verified (A260/A280 ≥ 1.9). Global m^6^A modification levels were quantified strictly following the manufacturer’s instructions for the EpiQuik™ m^6^A RNA Methylation Quantification Kit (Shanghai, China). The positive control was serially diluted with 1 × TE buffer to six concentration gradients ranging from 0.01 to 0.5 ng/µL for standard curve construction. After RNA binding, m^6^A-specific capture and antibody incubation steps, absorbance was measured at 450 nm, and the relative percentage of m^6^A modification was calculated via the kit-provided formula. All operations were performed on ice, with three biological replicates set for each group.

### 2.11. Immunohistochemical Staining

Tissue sections were placed in antigen retrieval solution (10 mM pH 6.0 citrate buffer containing 0.05% Tween-20) and boiled in a water bath for 20 min for antigen retrieval. After cooling to room temperature, sections were washed three times with PBS (5 min per wash). Tissue permeabilization was performed with 0.1% Triton X-100 for 15 min at room temperature, followed by sequential washing with PBS and sterile saline. After residual liquid was aspirated, goat serum was applied for 1 h at room temperature for blocking. Subsequent standard procedures including primary antibody incubation, secondary antibody incubation, DAB color development, hematoxylin counterstaining, gradient ethanol dehydration and neutral gum mounting were conducted. After slide drying, images were captured and analyzed under an inverted optical microscope.

### 2.12. Immunofluorescence

Cells were seeded into 24-well plates pre-laid with coverslips, fixed with 200 μL 4% paraformaldehyde per well for 15 min at room temperature, washed with PBS and stored in PBS at 4 °C for standby use. Blocking was conducted with 5% BSA (Sigma-Aldrich, St. Louis, MO, USA) for 1 h at room temperature. Primary antibodies (P-PI3K, 1:1000; P-AKT, 1:1000; BAX, 1:1000; BCL-2, 1:1000, Abcam, Cambridge, MA, USA) were incubated with samples overnight at 4 °C. After PBS washing, fluorescent secondary antibodies (anti-rabbit IgG, dilution 1:1000) were added and incubated under light protection for 1 h at room temperature. Samples were rinsed three times with PBST (5 min per rinse) and once with distilled water, followed by mounting with ProLong Diamond anti-fade mounting medium. Images were captured and analyzed under a fluorescence microscope.

### 2.13. Protein Molecule Docking

Standard UniProt entry IDs of target proteins were retrieved: ALKBH5 (Q6P6C2), PI3K (P42339), AKT (Q9Y243). Optimal three-dimensional crystal structures of the proteins were downloaded from the PDB database. Protein structures were uploaded to the HDOCK platform (http://hdock.phys.hust.edu.cn/, accessed on 12 May 2023) with flexible docking mode selected, generating the top 10 protein-binding conformations. The PyMOL (v3.1.6.1) software was used for preprocessing of protein 3D structures, including water molecule removal, hydrogen atom supplementation and Gasteiger charge assignment. Hydrogen bond networks (bond distance < 5 Å) and hydrophobic interactions between interacting proteins were visualized, and mapping diagrams of interacting amino acid residues and hydrogen bonds were plotted.

### 2.14. Statistical Methods

All experimental data were expressed as mean ± standard deviation (mean ± SD). Statistical analyses and graph plotting were performed using GraphPad Prism 9.0. For normally distributed quantitative data, an independent-samples *t*-test was adopted for two-group comparisons, and one-way analysis of variance (one-way ANOVA) for multi-group comparisons. For non-normally distributed quantitative data, the Wilcoxon rank-sum test was used for two-group comparisons, and the Kruskal–Wallis H test for multi-group comparisons. A *p*-value < 0.05 was considered statistically significant.

### 2.15. RNA-Seq and m^6^A-Seq

Testicular tissues from control and high-dose acetamiprid-exposed mice were subjected to RNA-seq and m^6^A-seq analysis (*n* = 3 biological replicates per group). Total RNA was extracted and evaluated by agarose gel electrophoresis and Qubit quantification before library construction. For m^6^A-MeRIP-seq, each sample was used to construct an m^6^A immunoprecipitation (IP) library and an input library. RNA was fragmented to approximately 100–200 nt, followed by immunoprecipitation with an m^6^A-specific antibody for the IP library, whereas the input library was processed without immunoprecipitation as the background control. Libraries were quality-checked and sequenced on the Illumina NovaSeq 6000 platform (Illumina, San Diego, CA, USA).

Raw reads were processed with fastp (v0.19.4) for adapter removal, poly-X tail trimming, and filtering of low-quality and short reads, and Q20, Q30, and GC content were assessed. Clean reads were aligned to the Mus musculus reference genome (Ensembl release 101) using HISAT2 (v2.2.1).

m^6^A-seq data were analyzed using exomePeak2 (v1.4.0) for peak identification and differential analysis, with input libraries used to account for background RNA abundance. m^6^A enrichment was assessed by scanning exonic regions and statistically comparing IP enrichment against input. For two-group comparison, IP and input data from both groups were jointly analyzed to assess changes in m^6^A enrichment. Differential m^6^A peaks were selected using |log_2_FC| ≥ 1 and *p* < 0.05. Peaks were annotated with ANNOVAR (2013Aug23) to determine associated genes and genomic regions, and motif analysis was performed with HOMER (v4.10) in RNA mode (-rna).

For RNA-seq, strand-specific transcript quantification was performed with StringTie (v2.2.1), and a gene-level count matrix was generated using the prepDE.py script. Differential expression analysis was performed with DESeq2 using median-of-ratios normalization, and DEGs were defined as genes with |log_2_FC| ≥ 1 and *p* < 0.05.

For integration, genes associated with significant differential m^6^A peaks (ANNOVAR (2013Aug23) annotation) were matched with DEGs. Genes meeting both m^6^A-seq (|log_2_FC| ≥ 1, *p* < 0.05) and RNA-seq (|log_2_FC| ≥ 1, *p* < 0.05) criteria were defined as jointly altered genes for subsequent integrated analyses. GO and KEGG enrichment analyses of genes associated with differential m^6^A peaks, DEGs, and jointly altered genes were performed using the relevant R packages in OmicStudio 3.23 based on the hypergeometric test, with *p* < 0.05 and Benjamini–Hochberg correction. GSEA (v4.3.2) was performed using gene expression data and predefined GO/KEGG gene sets, with |NES| > 1, nominal *p* < 0.05, and FDR q < 0.25 as significance criteria.

## 3. Research Results

### 3.1. Acetamiprid Exposure Induces Decreased Sperm Quality and Testicular Histopathological Injury in Mice

**Figure 1:**
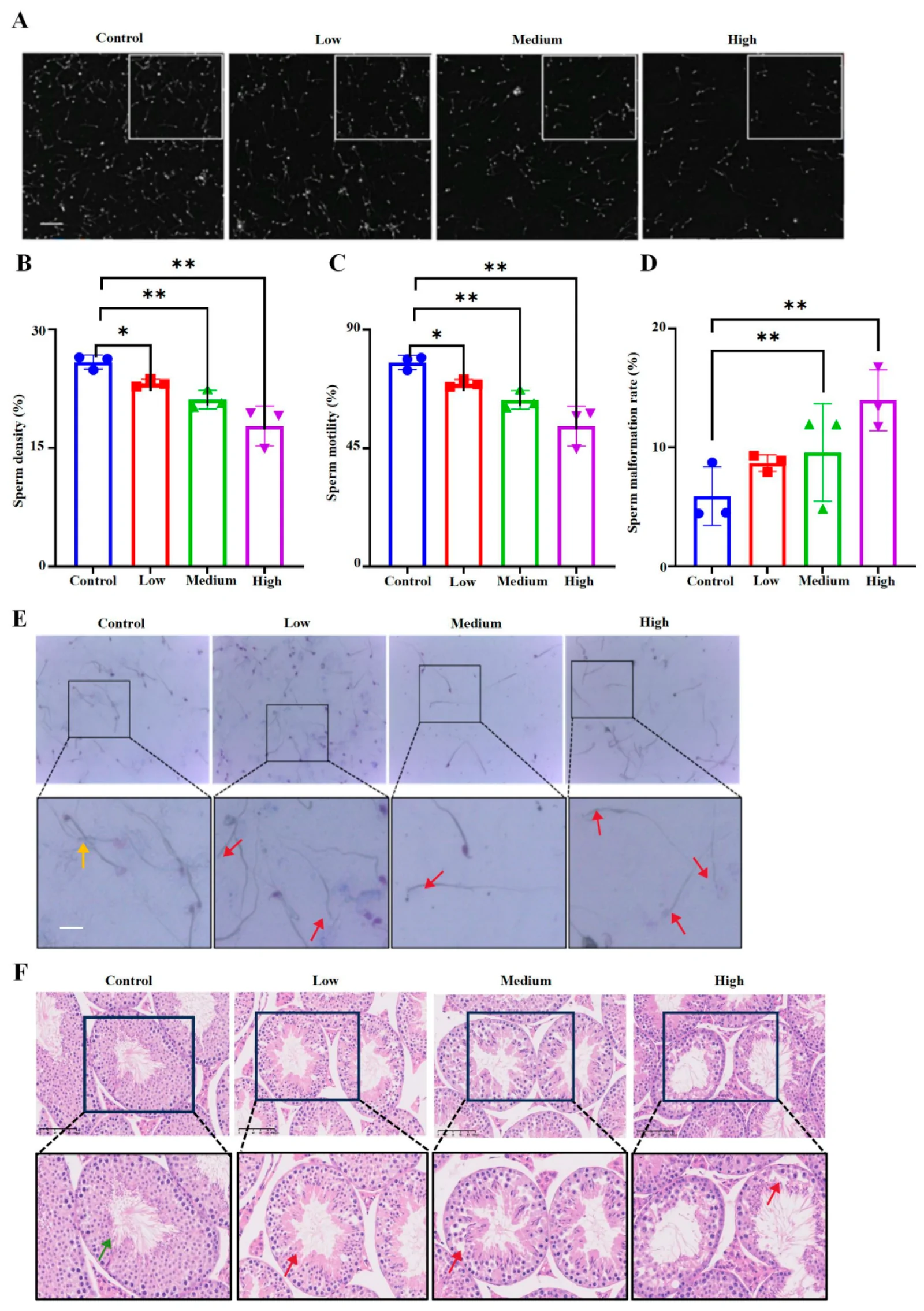
Effects of acetamiprid exposure on testicular tissue and sperm quality in C57BL/6J mice. (**A**) Changes in sperm density, motility and deformity rate in mice of different dose groups. (**B**) Statistical analysis results of sperm density. (**C**) Results of statistical analysis of sperm motility. (**D**) Statistical analysis results of sperm deformity rate. (**E**) Effects of acetamiprid exposure on sperm morphology in mice (20×). The yellow arrow indicates normal sperm. The red arrow indicates abnormal sperm. (**F**) HE staining showed pathological changes in testicular tissues of different groups (20×). Scale bars: 100 µm. The green arrow indicates normal spermatogenic cells; The red arrow indicates abnormal spermatogenic cells. The data are shown as the means ± SDs. The data are shown as the means ± SDs. * *p* < 0.05, ** *p* < 0.01, compared with the control group.

To characterize the male reproductive toxicity of ACE, 2-week-old male C57BL/6J mice were exposed via free drinking water for 6 months (low-, medium-, and high-dose groups at 4.55, 9.10, and 18.20 mg/kg bw/d, respectively), followed by assessment of sperm quality and testicular histomorphology. Sperm analysis revealed that, compared with the control group, the ACE-exposed groups exhibited a dose-dependent decrease in sperm concentration and total motility, with a corresponding dose-dependent increase in sperm malformation rate (Figure 1A–D). Diff-Quik staining of sperm morphology showed that spermatozoa in the control group displayed intact head contours, straight necks, and extended tails; in the ACE-exposed groups, morphological defects including headless sperm, bent necks, and coiled tails were observed, and the proportion of abnormal spermatozoa increased with increasing dose (Figure 1E).

Hematoxylin and eosin (HE) staining of testicular sections revealed that in the control group, the seminiferous tubule walls were intact, with Sertoli cells and germ cells at various stages orderly arranged in well-defined layers; in the ACE-exposed groups, disorganization of the seminiferous epithelium, shedding and reduction in germ cells, and increased cellular debris within the tubular lumens were observed, with the severity of lesions increasing with dose (Figure 1F). These results collectively demonstrate that chronic ACE exposure induces structural damage to the testicular seminiferous epithelium and impairs sperm quantity and quality at the functional level.

### 3.2. Acetamiprid Impairs the Structure and Function of TM3 Mouse Leydig Cells

**Figure 2:**
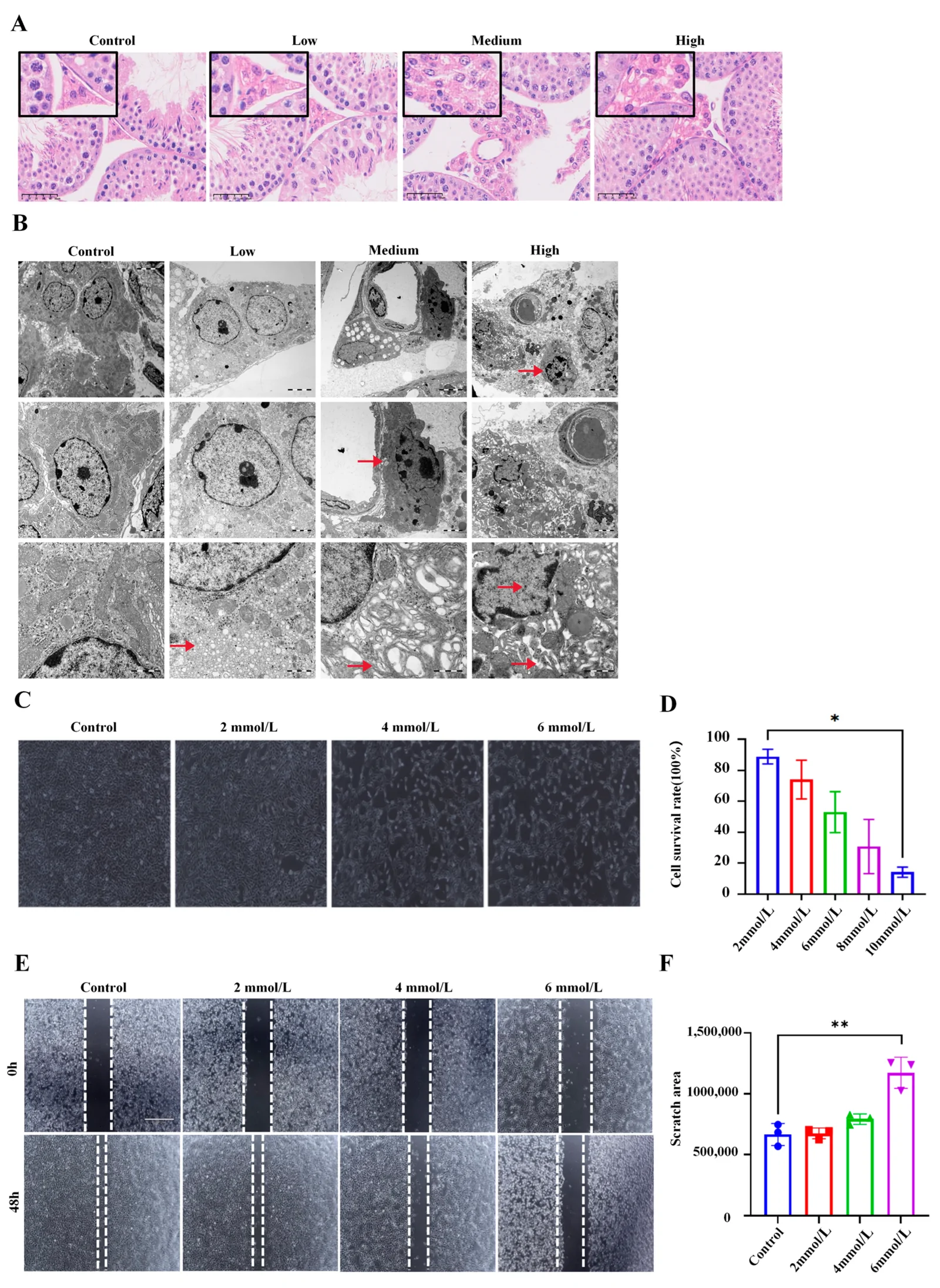
Structural and functional damage of testicular interstitial cells induced by acetamiprid exposure. (**A**) Morphological changes in TM3 cells exposed to different concentrations of acetamiprid (20×). Scale bars: 50 µm. (**B**) Ultrastructural alterations of TM3 cells in different acetamiprid dose groups (2500×). The red arrow points to the structural damage related to early apoptosis of TM3 cells after exposure to acetamiprid. (**C**) The status of TM3 cells exposed to different concentrations of acetamiprid. (**D**) Survival rates of TM3 cells exposed to different concentrations of acetamiprid. (**E**) Scratch test results of TM3 cells after exposure to acetamiprid. (**F**) Statistical analysis results of scratches on TM3 cells after exposure to acetamiprid. The data are shown as the means ± SDs. * *p* < 0.05, ** *p* < 0.01, compared with the control group.

Leydig cells support spermatogenesis through androgen synthesis and are important target cells for investigating testicular toxicity. HE staining showed that, with increasing dose, Leydig cells in the ACE-exposed groups exhibited disorganized arrangement and a significant reduction in number (Figure 2A). Transmission electron microscopy revealed early apoptotic features in Leydig cells in the exposure groups, including chromatin margination, nuclear membrane shrinkage, mitochondrial swelling, decreased electron density of the matrix, and dilation of the smooth endoplasmic reticulum, with the extent of changes being dose-dependent (Figure 2B).

In vitro, the mouse Leydig cell line TM3 was used to establish an ACE exposure model. The CCK-8 dose–response curve showed an IC_50_ of 6.17 mmol/L after 24 h of ACE treatment; therefore, 2, 4, and 6 mmol/L were selected for subsequent experiments (Figure S1). Compared with the control group, ACE-treated cells exhibited reduced viability and morphological changes including cell shrinkage, decreased adherent area, and widened intercellular spaces, with a large number of morphologically abnormal cells observed in the high-concentration group (Figure 2C,D). Wound healing assays showed that cell migration decreased in a dose-dependent manner after ACE treatment (Figure 2E,F).

These in vivo and in vitro findings demonstrate that ACE acts directly on TM3 cells, impairing their structural integrity and fundamental biological functions.

### 3.3. Acetamiprid Selectively Downregulates ALKBH5 and Elevates Global m^6^A Modification Levels

**Figure 3:**
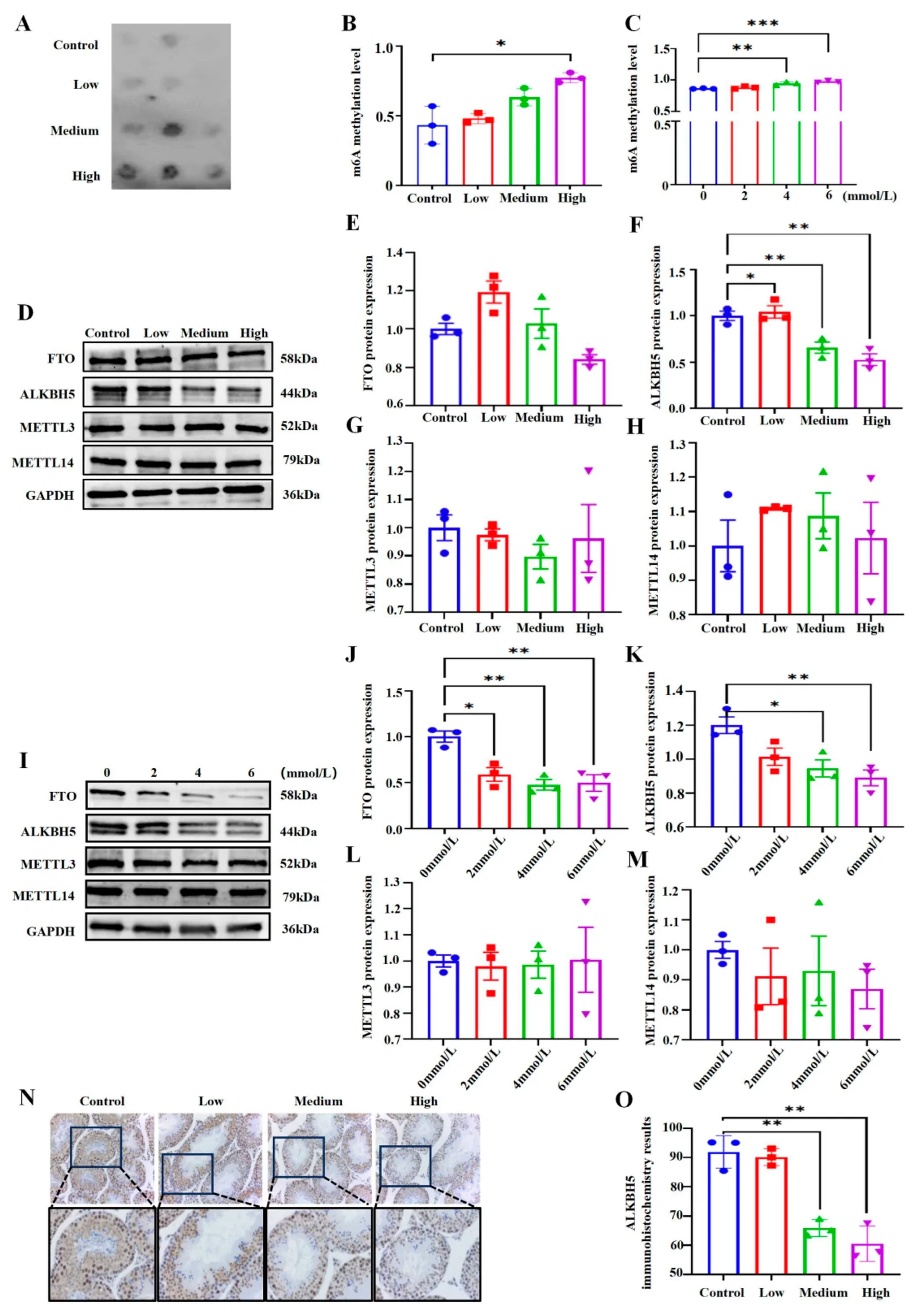
Effects of acetamiprid exposure on the overall RNA m^6^A methylation level and ALKBH5 expression. (**A**) Results of dot hybridization of mouse testicular tissue. (**B**) Statistical analysis results of the testicular tissue m6A experiment. (**C**) Statistical analysis results of m^6^A experiments on TM3 cells. (**D**) Testicular tissue FTO, ALKBH5, METTL3, and METTL14 immunoblot image. (**E**) Statistical analysis results of FTO Western blotting of testicular tissue. (**F**) Statistical analysis results of Western blotting of ALKBH5 in testicular tissue. (**G**) Statistical analysis results of METTL3 Western blotting in testicular tissue. (**H**) Statistical analysis results of METTL14 Western blotting in testicular tissue. (**I**) TM3 cells FTO, ALKBH5, METTL3 and METTL14 immunoblot expression images. (**J**–**M**) Statistical analysis of FTO, ALKBH5, METTL3, and METTL14 in TM3 cells by Western blot. (**N**) Immunohistochemistry was used to detect the expression and distribution of ALKBH5 protein in the testicular tissues of mice in different exposure groups. (**O**) Statistical analysis results of ALKBH5 protein expression levels in different exposure groups. The data are shown as the means ± SDs. * *p* < 0.05, ** *p* < 0.01, *** *p* < 0.001, compared with the control group.

To identify the upstream molecular mechanisms underlying ACE testicular toxicity, we first assessed m^6^A modification levels. Colorimetric quantification revealed that global m^6^A modification levels in total RNA from testicular tissues and TM3 cells increased significantly with increasing ACE dose in a dose-dependent manner (Figure 3A–C). To dissect the enzymatic basis of this change, we further examined the expression of m^6^A modification enzymes. In testicular tissues, METTL3, METTL14, and FTO protein levels showed no significant differences among groups, whereas ALKBH5 exhibited a dose-dependent downregulation (Figure 3D–H). In TM3 cells, METTL3 and METTL14 similarly showed no changes, while both ALKBH5 and FTO were significantly decreased (Figure 3I–M). Immunohistochemistry revealed that ALKBH5 staining intensity in the high-dose group was markedly diminished, with the most pronounced reduction observed in the seminiferous epithelial region (Figure 3N,O).

Given that ALKBH5 was the only m^6^A regulatory factor consistently downregulated in both in vivo and in vitro settings, and that its expression changes were consistent with the direction of global m^6^A elevation, we selected ALKBH5 as the target molecule for subsequent mechanistic studies.

### 3.4. Integrated Multi-Omics Analysis Suggests That m^6^A Modification Is Involved in TM3 Cell Apoptosis via the PI3K–AKT Pathway

**Figure 4:**
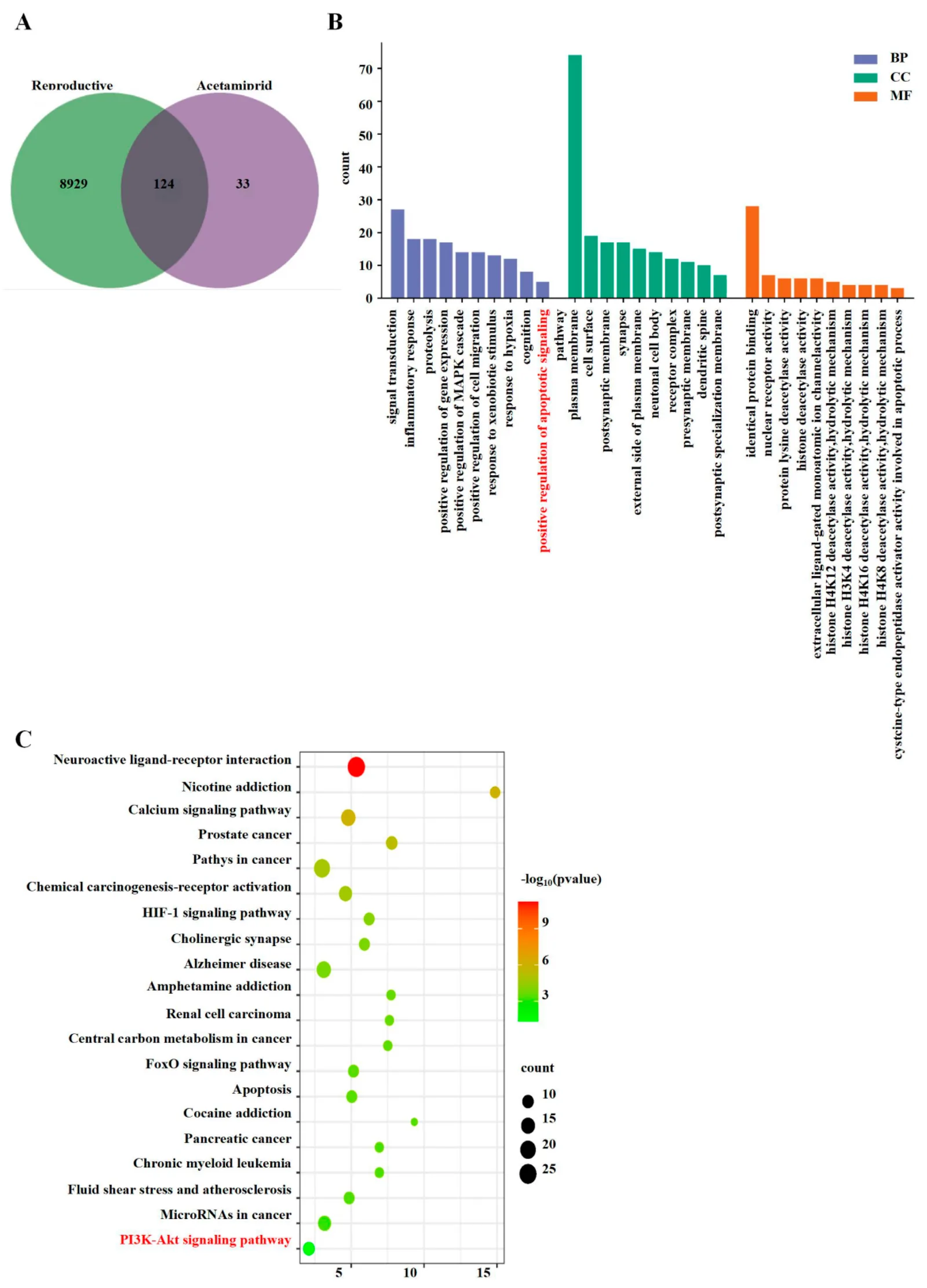
The results of network toxicology. (**A**) Venn diagram of the common targets of acetamiprid and reproductive damage. (**B**) GO enrichment analysis results of the target genes for reproductive damage induced by acetamiprid. (**C**) KEGG pathway enrichment analysis results of the target genes for reproductive damage induced by acetamiprid. The red text marks the apoptosis-related biological process and the PI3K-Akt signaling pathway focused on in this study.

To explore the downstream effector pathways of m^6^A homeostasis disruption, we employed a strategy combining network toxicology with high-throughput sequencing. A total of 157 potential targets of ACE were predicted based on the Swiss Target Prediction and SuperPred databases; 9053 reproductive toxicity-related genes were retrieved from the GeneCards and OMIM databases; and intersection of the two datasets yielded 124 candidate targets (Figure 4A). Gene Ontology (GO) enrichment analysis identified 216 biological processes, 49 cellular components, and 92 molecular function terms (FDR < 0.05), with the top 10 terms ranked by lowest FDR within each category showing significant enrichment of apoptosis-related processes (Figure 4B). KEGG analysis enriched a total of 112 pathways, among which the PI3K–AKT signaling pathway ranked among the top 20 pathways sorted by −log_10_P (Figure 4C), suggesting that ACE may drive apoptosis through inhibition of the PI3K–AKT pathway.

**Figure 5:**
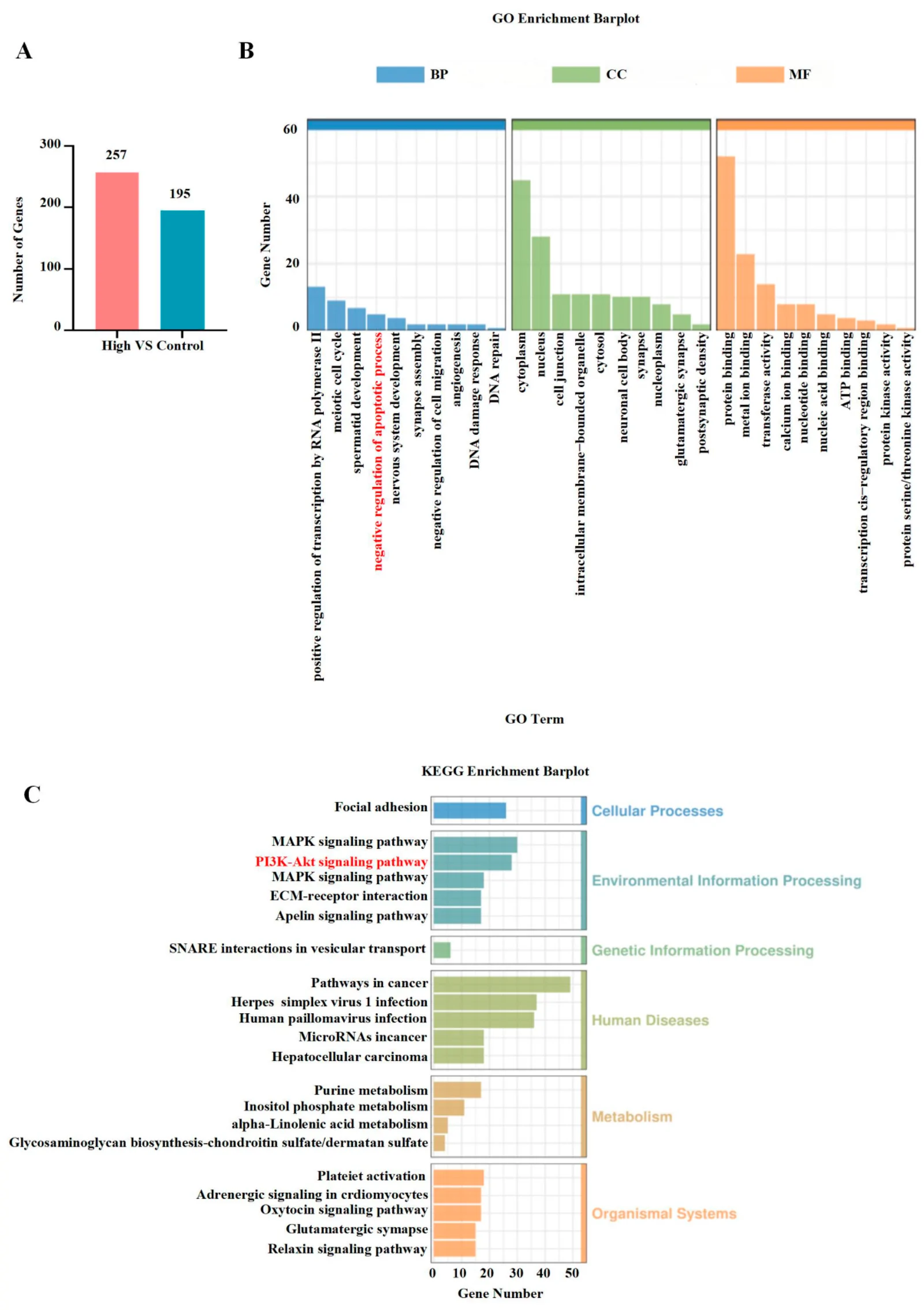
The results of transcriptome sequencing (RNA-seq). (**A**) Compared with the control group, the number of differentially expressed genes in the testicular tissue of mice exposed to acetamiprid. (**B**) Enrichment results of RNA-seq differential gene GO in testicular tissue of mice after exposure to acetamiprid. (**C**) Enrichment results of the KEGG pathway of RNA-seq differential genes in testicular tissue of mice after exposure to acetamiprid. The red text marks the apoptosis-related biological process and the PI3K-Akt signaling pathway focused on in this study.

**Figure 6:**
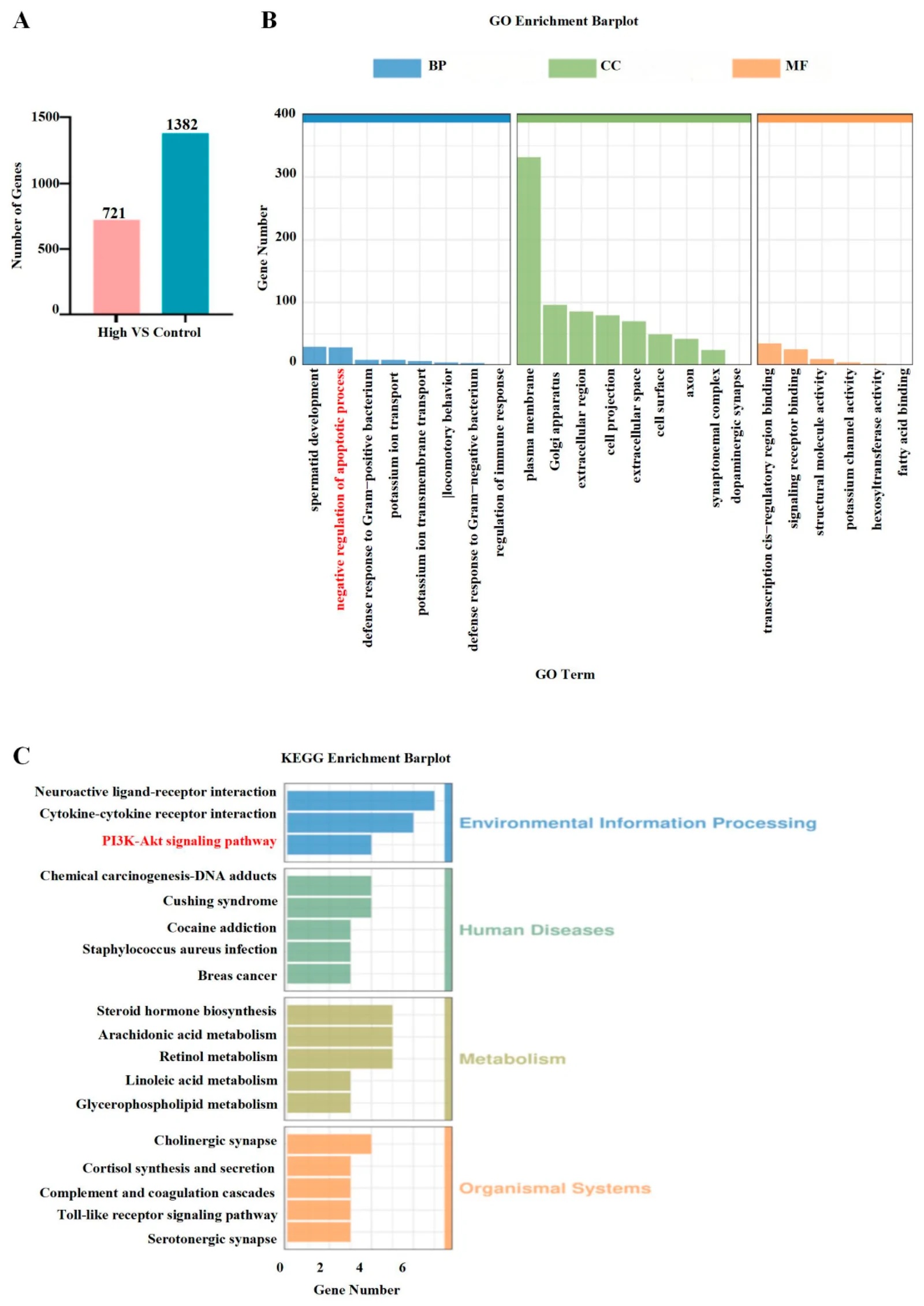
The results of m^6^A sequencing (m^6^A-seq). (**A**) Compared with the control group, the number of differentially methylated peaks in the testicular tissue of mice exposed to acetamiprid. (**B**) GO enrichment results of genes with differentially methylated peaks in testicular tissue of mice after exposure to acetamiprid. (**C**) Enrichment results of the KEGG pathway of the m6A-seq differential gene in testicular tissue of mice after exposure to acetamiprid. The red text marks the apoptosis-related biological process and the PI3K-Akt signaling pathway focused on in this study.

To validate the above predictions at the empirical level, testicular tissues from the control and high-dose groups were subjected to RNA-seq and m^6^A-seq. RNA-seq identified 452 differentially expressed genes (|log_2_FC| > 1, FDR < 0.05), of which 257 were upregulated and 195 were downregulated (Figure 5A); m^6^A-seq identified 2103 differentially methylated peaks, of which 721 showed increased modification levels and 1382 showed decreased levels (Figure 6A). GO and KEGG enrichment analyses of both datasets consistently pointed to apoptosis-related processes and the PI3K–AKT signaling pathway (Figure 5B,C and Figure 6B,C).

**Figure 7:**
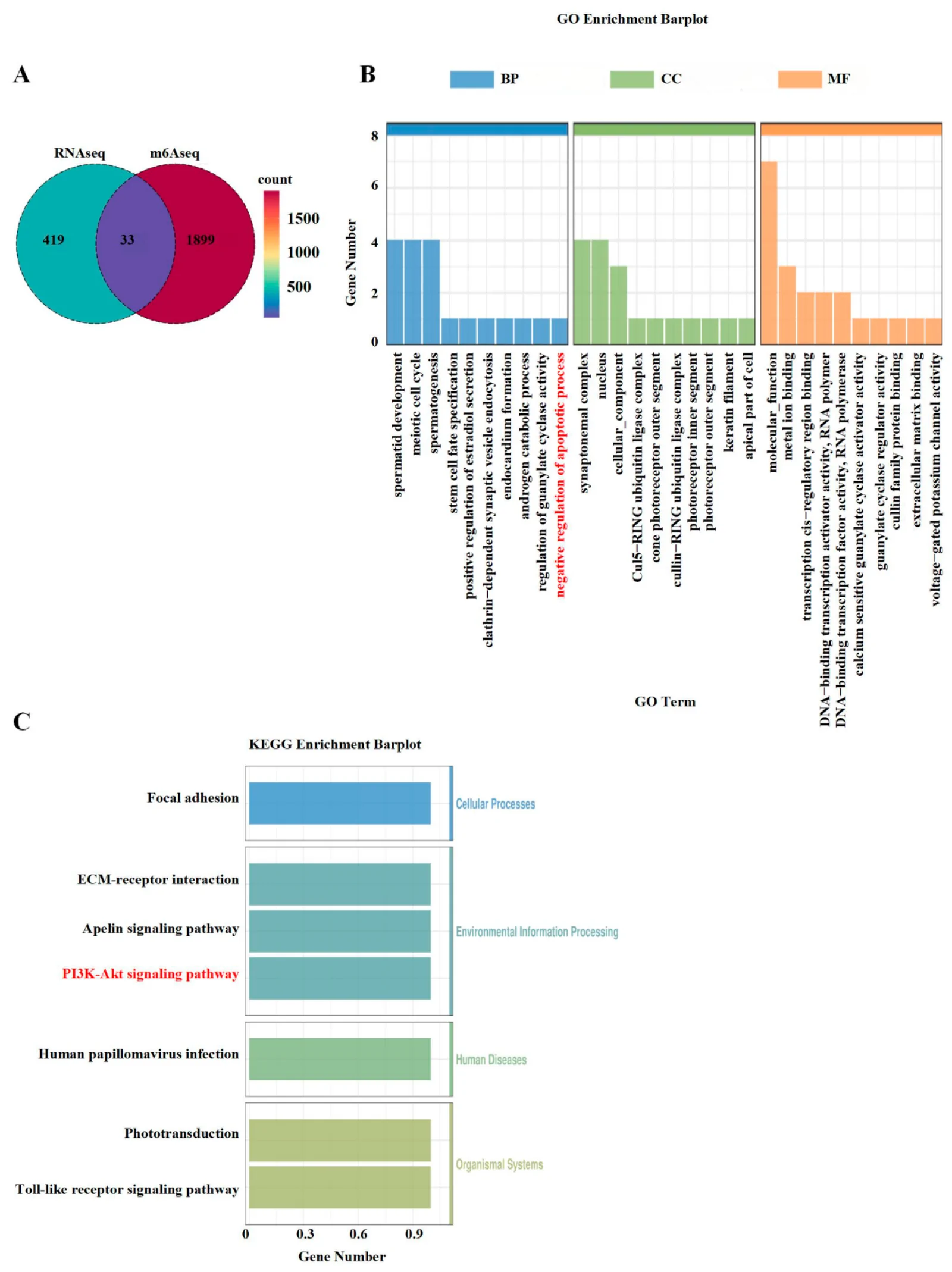
Combined analysis results of transcriptome sequencing (RNA-seq) and m^6^A sequencing (m^6^A-seq) of testicular tissue in mice exposed to acetamiprid. (**A**) Venn diagram of differentially expressed genes between RNA-seq and m^6^A-seq. (**B**) GO enrichment results of the intersection gene of RNA-seq and m^6^A-seq. (**C**) Enrichment results of the KEGG pathway of the intersection gene of RNA-seq and m^6^A-seq. The red text marks the apoptosis-related biological process and the PI3K-Akt signaling pathway focused on in this study.

Through the joint analysis of two datasets, we established a correlation between m^6^A modification and transcript abundance, yielding 33 co-variant genes that exhibited simultaneous significant changes in both m^6^A modification and mRNA expression levels (Figure 7A). This gene set was also significantly enriched in apoptosis regulatory functions and the PI3K–AKT pathway (Figure 7B,C).

Collectively, these results indicate that ACE exposure, while elevating global m^6^A levels, selectively alters the m^6^A modification status and concomitant expression of transcripts related to apoptosis and the PI3K–AKT pathway, suggesting that this pathway serves as a critical juncture through which m^6^A homeostasis disruption is involved in apoptosis.

### 3.5. Acetamiprid Inhibits PI3K–AKT Signaling and Activates Apoptotic Programs

**Figure 8:**
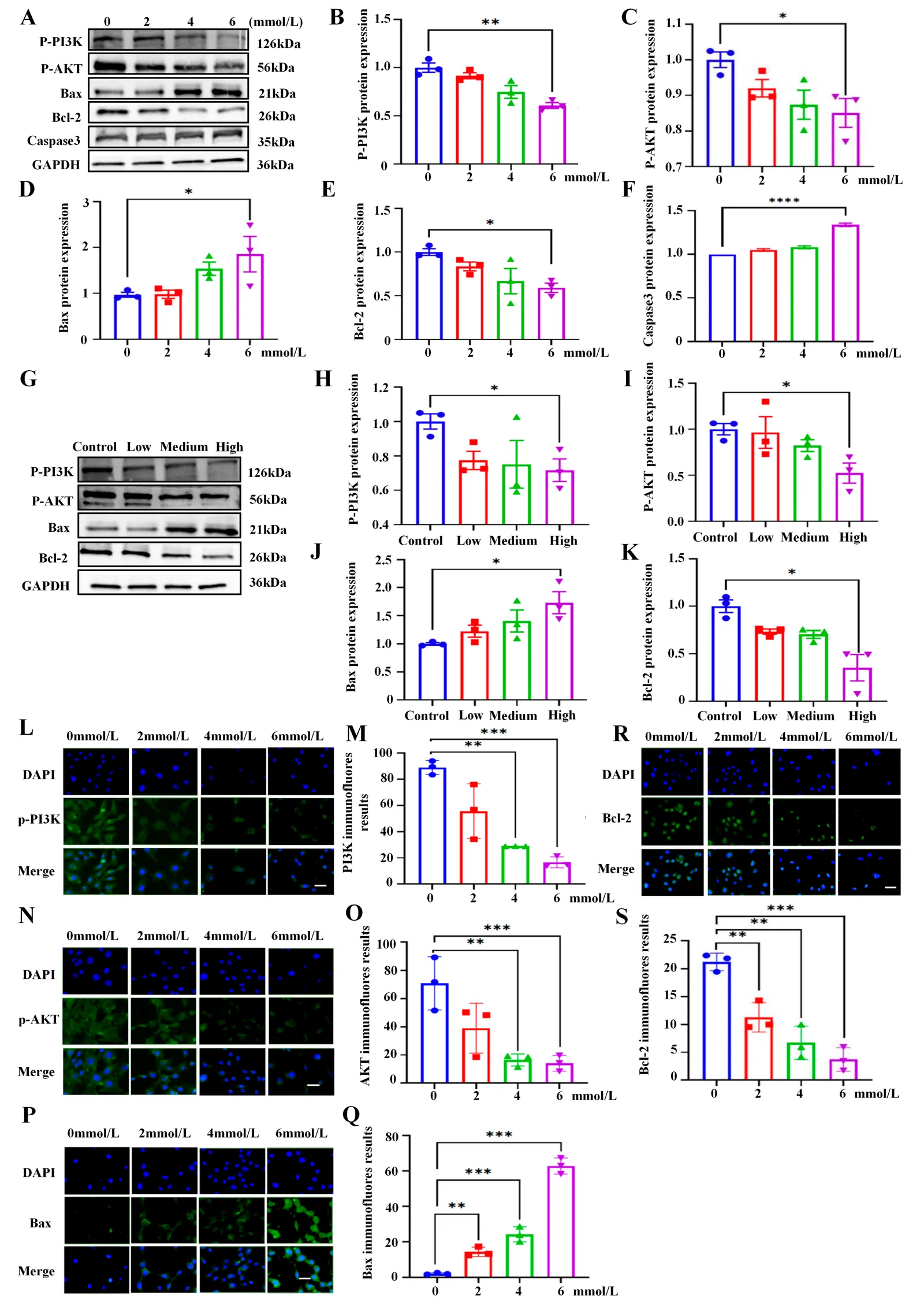
Exposure to acetamiprid alters the expression levels of apoptosis-related proteins and is involved in the expression of PI3K/AKT pathway proteins. (**A**) Western blotting images of P-PI3K, P-AKT, BAX, Bcl-2 and Caspase3 expressions in TM3 cells. (**B**–**F**) Statistical analysis results of Western blotting of P-PI3K, P-AKT, BAX, Bcl-2 and Caspase3 in TM3 cells. (**G**) Western blotting images of the expression of P-PI3K, P-AKT, BAX and Bcl-2 in testicular tissue. (**H**–**K**) Statistical analysis results of Western blotting of P-PI3K, P-AKT, BAX and Bcl-2 in testicular tissue. (**L**) Results of p-PI3K immunofluorescence staining. (**M**) Statistical analysis results of fluorescence intensity of p-PI3K protein. (**N**) Results of p-AKT immunofluorescence staining. (**O**) Statistical analysis results of fluorescence intensity of p-AKT protein. (**P**) BAX immunofluorescence staining results. (**Q**) Statistical analysis results of fluorescence intensity of BAX protein. (**R**) Results of Bcl-2 immunofluorescence staining. (**S**) Statistical analysis results of fluorescence intensity of Bcl-2 protein. The data are shown as the means ± SDs. * *p* < 0.05, ** *p* < 0.01, *** *p* < 0.001, **** *p* < 0.0001, compared with the control group.

We validated the aforementioned predictions by examining the expression levels of molecules involved in the PI3K–AKT pathway and apoptosis. Western blot analysis showed that, with increasing ACE dose, BAX and Caspase-3 expression in testicular tissues and TM3 cells was upregulated in a dose-dependent manner, while BCL-2, p-PI3K, and p-AKT expression was correspondingly decreased (Figure 8A–K). Immunofluorescence results were consistent with the protein quantification trends: BAX fluorescence intensity increased with increasing concentration, while BCL-2, p-PI3K, and p-AKT fluorescence signals progressively diminished (Figure 8L–S). These results demonstrate that ACE exposure disrupts the BCL-2/BAX balance and activates Caspase-3-dependent apoptotic pathways through inhibition of PI3K–AKT signaling activity, constituting the direct molecular basis for testicular tissue injury and TM3 cell apoptosis.

### 3.6. ALKBH5 Overexpression Restores PI3K–AKT Signaling Activity and Attenuates Acetamiprid-Induced Apoptosis

**Figure 9:**
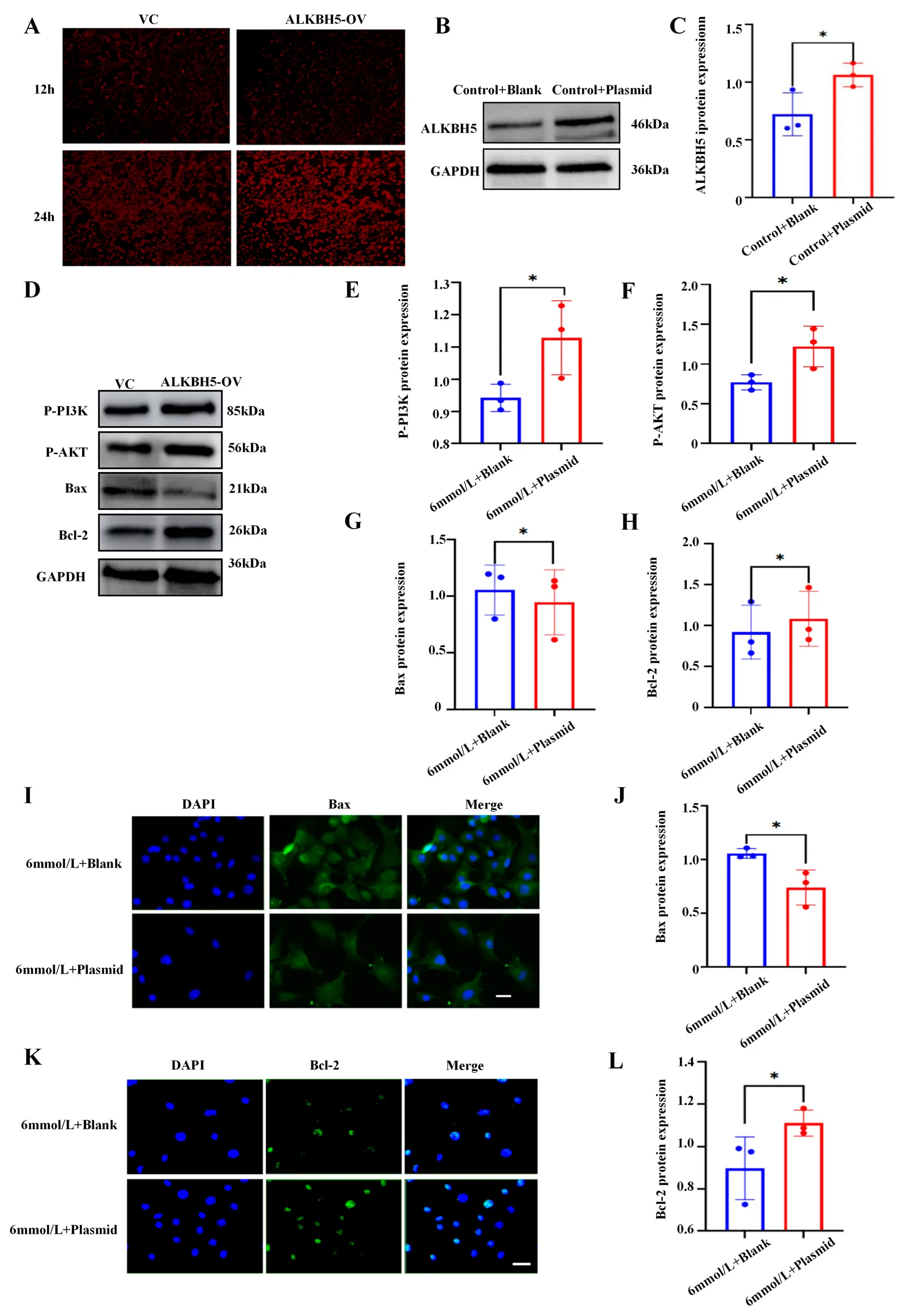
Overexpression of ALKBH5 can activate the PI3K/AKT signaling pathway and reverse apoptosis. (**A**) Observe the transfection efficiency of overexpressing ALKBH5 cells under fluorescence microscopy. (**B**) Western blot assay was used to detect the effect of acetamiprid exposure after overexpression of ALKBH5 on the expression level of ALKBH5 protein in TM3 cells. (**C**) Gray-scale statistical analysis of ALKBH5 protein. (**D**) Western blotting images of p-PI3K, p-AKT, Bax and Bcl-2 protein expression in overexpressed ALKBH5 cells. (**E**–**H**) Statistical analysis results of Western blotting of p-PI3K, p-AKT, Bax and Bcl-2 in overexpressed ALKBH5 cells. (**I**) Results of BAX immunofluorescence staining in cells overexpressing ALKBH5. (**J**) Statistical analysis results of fluorescence intensity of BAX protein. (**K**) Results of Bcl-2 immunofluorescence staining in cells overexpressing ALKBH5. (**L**) Statistical analysis results of fluorescence intensity of Bcl-2 protein. The data are shown as the means ± SDs. * *p* < 0.05, compared with the control group.

To establish the causal role of ALKBH5 in this signaling pathway, we generated a lentivirus-mediated stable ALKBH5 overexpression model in TM3 cells. Fluorescence microscopy showed that GFP-positive cells in all groups reached 90% at 24 h post-transfection (Figure 9A), indicating that lentiviral transduction was efficiently achieved under ACE treatment conditions; Western blot confirmed that ALKBH5 protein levels in the overexpression group were significantly higher than those in the control group (Figure 9B,C).

Rescue experiments showed that, compared with the ACE-alone treatment group, the ALKBH5 overexpression plus ACE treatment group exhibited significantly decreased BAX expression and significantly increased BCL-2, p-PI3K, and p-AKT levels (Figure 9D–L). Molecular docking results suggested a potential binding interface between ALKBH5 and PI3K/AKT (binding energies of −231.36 and −209.24 kcal/mol, respectively, Figure S2A–C); however, these results are computational predictions and their biological significance requires further verification.

These results indicate that ALKBH5 downregulation is associated with ACE-induced inhibition of PI3K-AKT signaling and activation of TM3 cell apoptosis, and that restoration of ALKBH5 levels can partially reverse this toxic effect.

## 4. Discussion

Acetamiprid (ACE), owing to its low vapor pressure, high insecticidal activity, and selectivity for insect nAChRs, has become one of the most widely used neonicotinoid insecticides globally. Its widespread residues in agricultural products and water bodies result in continuous internal exposure of the general population through dietary and drinking water routes. Previous animal studies have shown that developmental exposure to neonicotinoid insecticides can increase sperm malformation rates, germ cell apoptosis, and DNA fragmentation in male rats [19]. As an nAChR agonist, ACE can interfere with cholinergic signaling within the testis, acting on Sertoli cells and TM3 cells and affecting local neurotransmitter release, thereby disrupting the homeostasis of spermatogenesis and testosterone synthesis [20]. In addition, ACE can induce oxidative stress, inhibit steroid synthesis pathways, and reduce sperm concentration [21,22]. However, previous studies have largely focused on terminal events such as oxidative damage and apoptosis, with a lack of analysis regarding the upstream regulatory mechanisms that initiate these effects. In silico toxicological investigations have also raised the possibility that pesticides may disrupt male fertility through direct binding to reproduction-associated proteins [23]. Nevertheless, these existing lines of evidence still cannot fully interpret the molecular cascade underlying acetamiprid-provoked testicular injury, particularly from the epitranscriptomic perspective. In the present study, using a chronic drinking-water exposure model in mice, combined with a TM3 cell model and multi-omics analyses, we propose a molecular pathway in which ACE downregulates the m^6^A demethylase ALKBH5, elevates testicular m^6^A modification levels, inhibits PI3K-AKT signaling activity, and activates TM3 cell apoptosis, which is associated with testicular structural injury and decreased sperm quality. Similar to environmental heavy metal toxicants that induce male reproductive impairment, ACE exposure also triggers spermatogenic disorder and Leydig cell apoptosis. Nevertheless, distinct molecular regulatory patterns exist between pesticide-and heavy metal-mediated testicular damage. Heavy metal-evoked reproductive toxicity is frequently dominated by oxidative stress-driven DNA damage [24], whereas our findings highlight epitranscriptomic m6A dysregulation as a key initiating event underlying acetamiprid-provoked testicular injury.

The testis is the sole site of spermatogenesis, and the structural integrity of the seminiferous epithelium is a prerequisite for normal sperm production. In this study, ACE exposure caused decreased sperm concentration and motility and increased malformation rates, accompanied by disorganization of the seminiferous epithelium and reduction in germ cells, with the severity of damage increasing with dose, consistent with the findings of Toghan [25]. TM3 cells are located in the interstitium between seminiferous tubules and maintain the local endocrine microenvironment required for spermatogenesis through testosterone synthesis [26], and have been confirmed to be direct targets of ACE [27]. The present study showed that after ACE exposure, TM3 cells exhibited disorganized arrangement and reduced numbers, with early apoptotic features including chromatin margination, nuclear membrane shrinkage, and dilation of smooth endoplasmic reticulum observed under electron microscopy, while in vitro assays demonstrated inhibited cell viability and migration. Based on these observations, we hypothesize that TM3 cell injury and reduction may weaken their support for the spermatogenic microenvironment, thereby indirectly exacerbating germ cell apoptosis and impaired sperm production. However, as we did not measure serum and intratesticular testosterone levels or the expression of key steroidogenic enzymes (such as StAR, CYP11A1, and 3β-HSD), the contribution of “endocrine microenvironment imbalance indirectly impairing spermatogenesis” remains inferential at this stage and requires further verification.

Environmental chemicals can affect gene expression and cellular function through epigenetic modifications [27,28]. m^6^A is the most abundant internal modification in eukaryotic mRNA, dynamically regulated by the “writer–eraser–reader” system, and its homeostasis disruption is associated with various diseases [29], playing critical roles at each stage of mammalian spermatogenesis [30,31]. The present study found that ACE exposure significantly elevated global m^6^A levels in testicular tissues. Among the m^6^A modification enzymes, METTL3 and METTL14 showed no changes in either in vivo or in vitro settings; ALKBH5 was consistently downregulated in both testicular tissues and TM3 cells, while FTO was decreased only in TM3 cells. Given that ALKBH5 was the only regulatory factor consistently downregulated in both in vivo and in vitro contexts, and that its expression change was consistent with the direction of global m^6^A elevation, we propose that ALKBH5 downregulation is the primary enzymatic basis for ACE-induced m^6^A hypermodification. The tissue–cell difference in FTO expression may arise from differences in subcellular localization, substrate preferences, and cellular composition heterogeneity of testicular tissues. Notably, the elevation of global m^6^A levels measured by colorimetric assay is not contradictory to the observation in m^6^A-seq that the number of peaks with decreased modification exceeded that of increased peaks: the former reflects stoichiometric changes at the total RNA level, while the latter reflects redistribution at the site-specific level, representing different dimensions of measurement. This inference is consistent with the known reproductive functions of ALKBH5: Alkbh5-deficient mice exhibit increased germ cell apoptosis, spermatogenic arrest, and testicular atrophy due to aberrant splicing and 3′UTR processing defects in meiotic transcripts [32,33]. Thus, ALKBH5 downregulation provides an interpretable upstream node for ACE reproductive toxicity.

The PI3K–AKT pathway is a critical hub for cellular metabolism, proliferation, and survival [34,35], and m^6^A modification has been demonstrated to regulate its activity by altering the stability or translational efficiency of pathway-related transcripts [36]. In this study, integrating network toxicology, RNA-seq, and m^6^A-seq, we found that differentially expressed genes, differentially methylated genes, and their co-variant gene sets were all enriched in apoptosis regulatory functions and the PI3K–AKT pathway; protein-level analyses further confirmed that after ACE exposure, p-PI3K and p-AKT were decreased, the BCL-2/BAX ratio was reduced, and Caspase-3 activation was enhanced. These findings are consistent with previous reports that m^6^A-modified transcripts are enriched in the PI3K–AKT pathway, and that hypermethylation of pathway-related genes is often accompanied by apoptosis activation [37]; similar m^6^A–PI3K/AKT coupling has also been observed in models of anthracycline toxicity and in HT-29 cells [38]. Collectively, PI3K–AKT signaling inhibition constitutes a critical intermediate link connecting m^6^A homeostasis disruption to TM3 cell apoptosis. Notably, the coordinated changes in the BCL-2/BAX ratio, Caspase-3 activation, PI3K-AKT signaling, ALKBH5 expression, and m6A modification levels indicate that the observed apoptosis involves a defined molecular mechanism rather than nonspecific cytotoxicity at high acetamiprid concentrations.

The possibility that the testicular effects are secondary to systemic toxicity should also be considered. Nevertheless, the dose-dependent testicular damage and the direct effects observed in TM3 cells suggest that the testicular effects are unlikely to be solely secondary to systemic toxicity. This also highlights the need for future studies to monitor systemic parameters throughout the entire exposure period, in order to further distinguish direct testicular toxicity from systemic contributions.

ALKBH5 participates in embryonic development, tumor progression, and immune responses through dynamic removal of m^6^A from target RNAs [39,40], and its deficiency impairs germ cell development and spermatogenesis [41]; however, whether environmental pollutants affect male reproductive function through ALKBH5 has rarely been reported previously. The functional rescue experiments in this study demonstrated that ALKBH5 overexpression restored p-PI3K and p-AKT levels, reduced BAX expression, and alleviated ACE-induced apoptosis, functionally supporting that ALKBH5 is positioned upstream of this toxicity pathway. Similar phenomena have been observed for other pollutants: polycyclic aromatic hydrocarbons can disrupt m^6^A modification and impair cellular function by reducing ALKBH5 expression [42]. Molecular docking results suggested a potential binding interface between ALKBH5 and PI3K/AKT; however, given that ALKBH5 functions in RNA demethylation, its regulation of PI3K–AKT is more likely achieved through altering the m^6^A modification status of transcripts encoding pathway components. Therefore, the docking results should be regarded only as suggestive clues, and whether direct protein–protein interactions exist requires further experimental verification. Moreover, the mechanism by which ACE downregulates ALKBH5—whether through nAChR-mediated cholinergic signaling, oxidative stress-related transcriptional regulation, or post-transcriptional protein stability pathways—remains an unresolved upstream question that could be tested using nAChR antagonists or antioxidant interventions.

Several limitations of this study should be acknowledged. First, the in vitro model employed TM3 cells, which cannot recapitulate the paracrine interactions among Sertoli cells, germ cells, and macrophages within the testis. Second, while our integrated RNA-seq and m^6^A-seq analysis identified 33 co-variant genes enriched in the PI3K-AKT pathway, we acknowledge that global m^6^A quantification alone cannot pinpoint the specific transcripts mediating PI3K/AKT inhibition. MeRIP-qPCR validation of individual PI3K/AKT-related transcripts would provide more direct evidence, and this will be addressed in future studies. Third, although ALKBH5 overexpression rescue experiments demonstrated that restoring ALKBH5 attenuated ACE-induced m^6^A elevation, PI3K/AKT inhibition, and apoptosis, loss-of-function experiments such as ALKBH5 knockdown or knockout were not performed in this revision. Future studies employing ALKBH5 knockdown or knockout models will be needed to further establish the causal role of the ALKBH5–m^6^A–PI3K/AKT axis in ACE-induced testicular injury. It should be noted that the doses used in the present 6-month mouse model (4.55, 9.10, and 18.2 mg/kg/day) and the in vitro concentrations (0, 2, 4, and 6 mmol/L) were selected for mechanistic hazard exploration and are substantially higher than environmental exposure levels through vegetables and fruits as well as general human exposure levels; therefore, these results cannot be directly extrapolated to human risk assessment. In addition, TM3 cells are a mouse Leydig cell line and may not fully reflect human Leydig cell characteristics, and species differences should also be considered. Nevertheless, our findings provide hazard evidence that sustained acetamiprid exposure can trigger epigenetic dysregulation and testicular injury in male mammals, with warning implications for occupationally exposed populations.

In conclusion, this study demonstrates that ACE exposure induces testicular m^6^A modification imbalance through downregulation of ALKBH5, which in turn inhibits PI3K–AKT signaling and activates TM3 cell apoptosis, which is associated with testicular structural damage and decreased sperm quality. These findings extend the understanding of the mechanisms underlying neonicotinoid pesticide reproductive toxicity from the perspective of RNA epitranscriptomics, suggest that ALKBH5 may serve as a candidate molecular marker and potential intervention node for pesticide reproductive toxicity evaluation, and provide a molecular mechanistic basis for health risk assessment of environmental pollutants.

## 5. Conclusions

Combining in vivo animal assays, in vitro cellular functional experiments and integrated multi-omics analysis, the present study confirms that acetamiprid exposure adversely affects sperm quality and TM3 cells in male mice, leading to testicular injury. The core molecular mechanism underlying such reproductive toxicity centers on the downregulated expression of demethylase ALKBH5. Reduced ALKBH5 abundance elevates global m^6^A modification levels, suppresses PI3K-AKT signaling cascade activity, and consequently contributes to apoptosis of TM3 cells. Functional rescue assays via ALKBH5 overexpression in vitro were capable of fully reversing all toxic phenotypes induced by acetamiprid. Collectively, our findings provide epigenetic evidence for environmental health risk assessment of acetamiprid, and lay a molecular mechanistic foundation for studies targeting the intervention of pesticide-caused male reproductive toxicity.

## Data Availability

The data will be made available upon request. Please contact the corresponding authors for additional details.

## Supplementary Materials

The following supporting information can be downloaded at https://www.mdpi.com/article/10.3390/toxics14100903/s1, Figure S1: Effect of acetamiprid on TM3 cell viability. TM3 cells were treated with acetamiprid (0–10 mmol/L) for 24 h. Cell viability was determined by the CCK-8 assay. Data are shown as the mean ± SD (*n* = 3). The calculated IC_50_ is 6.17 mmol/L; Figure S2: Molecular docking analysis results of ALKBH5 with PI3K and AKT. (A) Three-dimensional structure of the ALKBH5-PI3K interaction and a magnified view of the binding interface. (B) Three-dimensional structure of the ALKBH5-AKT interaction and a magnified view of the binding interface. (C) The binding energy scores corresponding to the protein–protein docking of the two pairs.
