# Supplementary material for: The Toxic Effects and Mechanism of Acetamiprid Exposure on Testicular Tissue: ALKBH5 Regulates m6A Modification to Affect PI3K-AKT-Mediated Apoptosis
Source: Toxics. 2026 Oct 12;14(10):903. doi: 10.3390/toxics14100903 (PMC13709516; doi:10.3390/toxics14100903)
Supplement: Supplementary file 1 [file toxics-14-00903-s001.zip › toxics-4571348-supplementary.pdf]

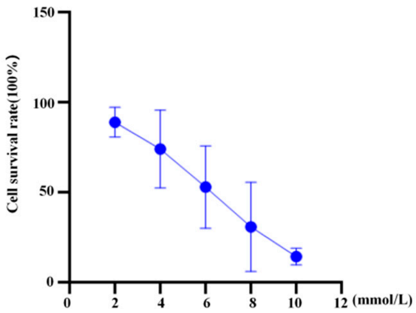

Figure S1 Effect of acetamiprid on TM3 cell viability. TM3 cells were treated with acetamiprid (0–10 mmol/L) for 24 h. Cell viability was determined by the CCK-8 assay. Data are shown as the mean ± SD (n = 3). The calculated IC_{50} is 6.17 mmol/L.

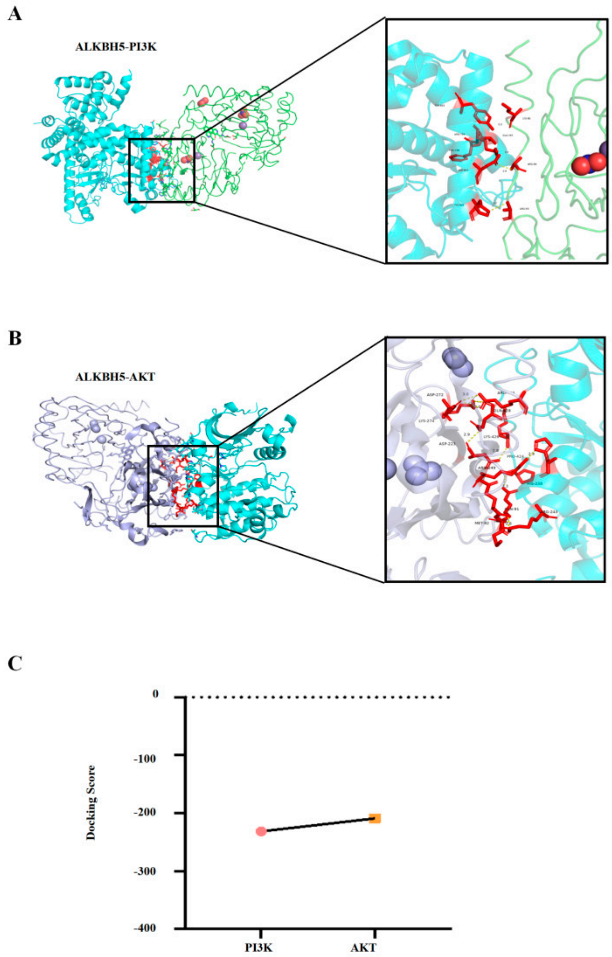

Figure S2 Molecular docking analysis results of ALKBH5 with PI3K and AKT. (A) Three-dimensional structure of the ALKBH5-PI3K interaction and a magnified view of the binding interface. (B) Three-dimensional structure of the ALKBH5-AKT interaction and a magnified view of the binding interface. (C) The binding energy scores corresponding to the protein－protein docking of the two pairs.
